# Signaling pathway of targeting the pancreas in the treatment of diabetes under the precision medicine big data evaluation system

**DOI:** 10.3389/fgene.2023.1119181

**Published:** 2023-02-17

**Authors:** Ge Song, Yiqian Zhang, Yihua Jiang, Huan Zhang, Wen Gu, Xiu Xu, Jing Yao, Zhengfang Chen

**Affiliations:** ^1^ Department of Endocrinology, Soochow University Affiliated Changshu First People’s Hospital, Changshu, Jiangsu, China; ^2^ Department of Endocrinology, Changshu Hospital Affiliated to Soochow University, Changshu No.1 People’s Hospital, Medical College of Soochow University, Changshu, Jiangsu, China

**Keywords:** diabetes disease, pancreatic target, precision medicine big data, signal path, targeting the pancreas

## Abstract

Diabetes is a chronic noncommunicable disease, which is related to lifestyle, environmental and other factors. The main disease of diabetes is the pancreas. Inflammation, oxidative stress and other factors can interfere with the conduction of various cell signaling pathways, thus inducing pancreatic tissue lesions and diabetes. Precision medicine covers epidemiology, preventive medicine, rehabilitation medicine and clinical medicine. On the basis of precision medicine big data analysis, this paper takes pancreas as the target to analyze the signal pathway of diabetes treatment. This paper analyzes from the five aspects of the age structure of diabetes, the blood sugar control standard of type 2 elderly diabetes mellitus, the changes in the number of diabetic patients, the ratio of patients using pancreatic species and the changes in blood sugar using the pancreas. The results of the study showed that targeted pancreatic therapy for diabetes reduced the diabetic blood glucose rate by approximately 6.94%.

## 1 Introduction

The organic combination of precision medicine and big data technology is conducive to the analysis and sharing of medical data, so as to better meet the needs of medical development and lay a solid foundation for the development of related medical research. Diabetes is an endocrine inflammatory disease. At present, the treatment presents the characteristics of multiple pathways and multiple targets. The pancreas is an important target for diabetes treatment. The application of precision medicine big data to the research on the treatment of diabetes with the pancreas as the target is conducive to promoting the progress of diabetes medicine.

The incidence of diabetes is getting higher and higher, and many scholars have studied it. Bensellam M presented the identified molecular mechanisms involved in the dedifferentiation of cells in the diabetic pancreas that produce and release the hormone insulin. The roles and inhibitors of differentiation proteins were discussed and the emerging role of non-coding RNAs (Ribonucleic Acid) was highlighted (Bensellam et al., 2018). Tuttolomondo A studied several factors that affect overall cerebrovascular risk to varying degrees in people with diabetes. Diabetes may lead to more insidious brain damage represented by lacunar infarcts, which would increase the risk of dementia and lead to a dramatic decline in cognitive function (Tuttolomondo, 2018). Hu C believed that the decreased ability of insulin to promote the processing and storage of glucose in muscle is due to impaired activation of glycogen synthase. Decreased glucose storage may occur due to decreased glucose uptake, and safety has been cognitively impaired secondary (Hu and Jia, 2018). In the Cardiovascular Health Study, Cho N H assessed the relationship between patients with subclinical cardiovascular disease, patients with diabetes and impaired glucose tolerance and normal subjects and the risk of clinical vascular disease (Cho et al., 2018). Liu believed the prevalence of diabetic lesions is high, and these complications are often associated with poor medication adherence and uncontrolled diabetes. The aim of the study was to determine medication adherence in patients with uncontrolled diabetes and to compare characteristics and identified barriers between patients with good and poor adherence to medication (Liu et al., 2018). Lane W mentioned that people with diabetes are prone to foot ulcers. If these ulcers do not heal, the patient may also undergo foot amputation, with diabetes preventing postoperative wound healing (Lane et al., 2017). Feig D S investigated whether microalbuminuria predicts later development of increased proteinuria and early mortality in patients with type 2 diabetes. Morning urine samples from diabetes clinic patients aged 50–75 years were examined by radioimmunoassay (Feig et al., 2018). Although there are many studies on the theory of diabetes, further research is needed on the treatment of diabetes.

The pancreas-targeted treatment of diabetes is widely used in medicine. Research by Marathe P H demonstrated that permanent neonatal diabetes may be caused by a complete lack of glucokinase activity. It reported three new cases of glucokinase-related permanent neonatal diabetes. Autosomal recessive inheritance and enzyme deficiencies are typical features of inborn errors of metabolism that occur in the glucose-insulin signaling pathway in these subjects (Marathe et al., 2017). Ogurtsova K explored the etiology of diabetes-related cognitive decline. The etiology involves insulin receptor downregulation, neuronal apoptosis, and glutamatergic neurotransmission (Ogurtsova et al., 2017). Bragg F studied the role of signal transduction and transcriptional activator protein signaling pathway in autoimmune diabetes (Bragg et al., 2017). Rowan J A provided a new strategy for diabetes prevention and treatment by studying the vascular endothelial cell nuclear factor signaling pathway in diabetes (Rowan et al., 2018). Wang Q mentioned that diabetes impairs the mobilization of hematopoietic stem cells from the bone marrow, thereby worsening the outcome of hematopoietic stem and progenitor cell transplantation and diabetic complications (Wang et al., 2017). Willeit explored the potential effects of curcumin on cardiomyocyte hypertrophy, possible mechanisms of nuclear transcription factor signaling in diabetes, hyperglycemia- and insulin-induced cardiomyocyte hypertrophy, and antihypertrophic effects of curcumin in primary culture (Willeit et al., 2017). Chamberlain JJ mentioned that insulin acutely controls metabolism in adipocytes, but also nuclear transcription *via* proline-directed serine/threonine kinase-mediated “mitotic” signaling (Chamberlain et al., 2017). Although pancreas-targeted therapy for diabetes is widely used in medicine, there are still problems in its application.

By combining computer technology with medicine, new knowledge can be discovered and new modalities of diagnosis and treatment can be created. There have been many research reports on diabetes signaling pathways. This article took pancreatic islets as the target, and systematically discussed the related signaling pathways. At the same time, through the use of precision medicine big data analysis, some complex information was processed, so as to realize the diagnosis and prediction of diabetes.

## 2 Concepts related to precision medicine big data, diabetes and pancreas

### 2.1 Precision medicine big data

**FIGURE 1:**
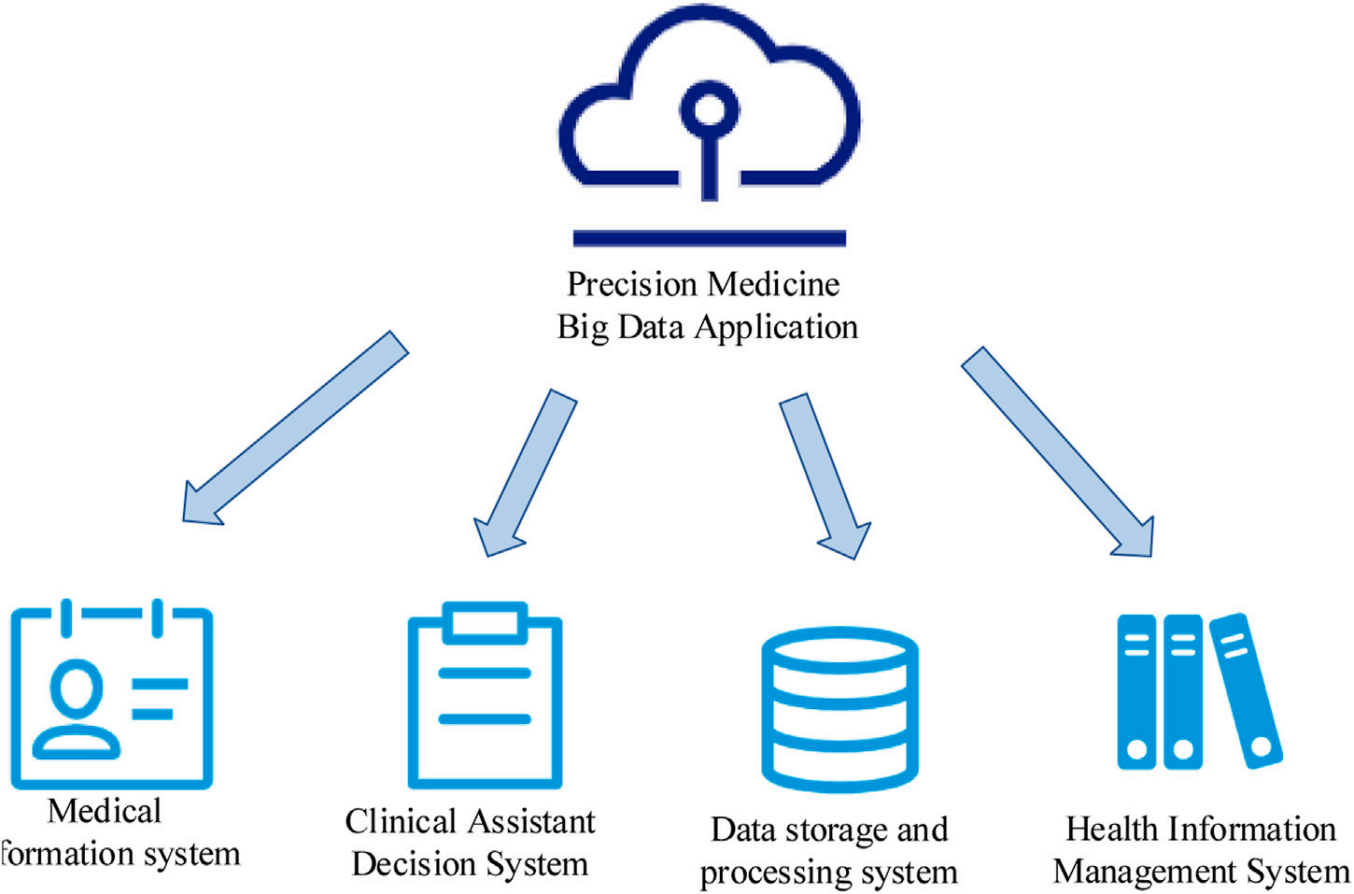
Big data applications in precision medicine.

Precision medicine is an upgraded version of personalized medicine. According to the biological characteristics of patients, especially the data of genomics, and through modern technology, it provides accurate prevention, diagnosis and treatment for the clinic. It can not only improve the treatment effect, but also prevent inappropriate treatment, excessive treatment, waste of resources and other phenomena, and save medical expenses (Riddell et al., 2017). The implementation of precision medicine is based on the development of gene sequence detection technology, and on the basis of network and big data technology. This technology is able to treat patients with minimal medical damage and minimal medical costs, and it not only restores their physical functions, but also ensures their mental health. The big data application of precision medicine is shown in Figure 1.

#### 2.1.1 Medical information system

In fact, medical information systems did not appear in recent years, but because of the complexity of medical services and the small coverage of medical information systems, they have not been widely used. There is a large amount of different medical data in different institutions, systems or databases. The challenge is how to integrate these data in an orderly manner without violating existing laws, regulations, and ethics; and how to conduct automatic computer analysis and mining without violating existing laws, regulations, and ethics. The construction and application of a high-quality medical information system is still an important prerequisite for the production of big data, and it is also an area where companies in the industry invest the most.

#### 2.1.2 Clinical auxiliary decision-making system

Big data is not to replace experienced doctors, but to allow more doctors to make high-quality judgments before they have enough experience, which is also an important role of precision medicine. This technology can help doctors diagnose and treat patients’ illnesses by analyzing various medical data such as patients’ medical records, test results, medical images, etc.

#### 2.1.3 Data storage and processing system

The development of the big data industry is inseparable from the support of infrastructure, and many companies have developed their own storage and processing platforms. However, the mainstream applications of software services are still on basic communication software such as cloud mailboxes and communication platforms. More software services in the medical field are also needed to improve the research and develop efficiency of pharmaceutical companies and strengthen the management of patients and clinical data by physicians.

#### 2.1.4 Health information management system

At present, many health management systems have emerged in mobile health management, such as diabetes management, sleep quality management, and intestinal health management. In fact, they all rely on a large number of data processing and high-quality sensors to realize real-time monitoring of data, but many systems have no way to intervene in time when dealing with exceptions. However, big data is like a chicken-and-egg process. In the face of massive data, the correlation would gradually become prominent, and corresponding auxiliary decision-making would emerge. Big data technology can provide a health information management platform with information collection, analysis and processing functions by collecting various medical data files of patients in medical institutions and sharing various medical data through network technology.

### 2.2 Diabetes

**FIGURE 2:**
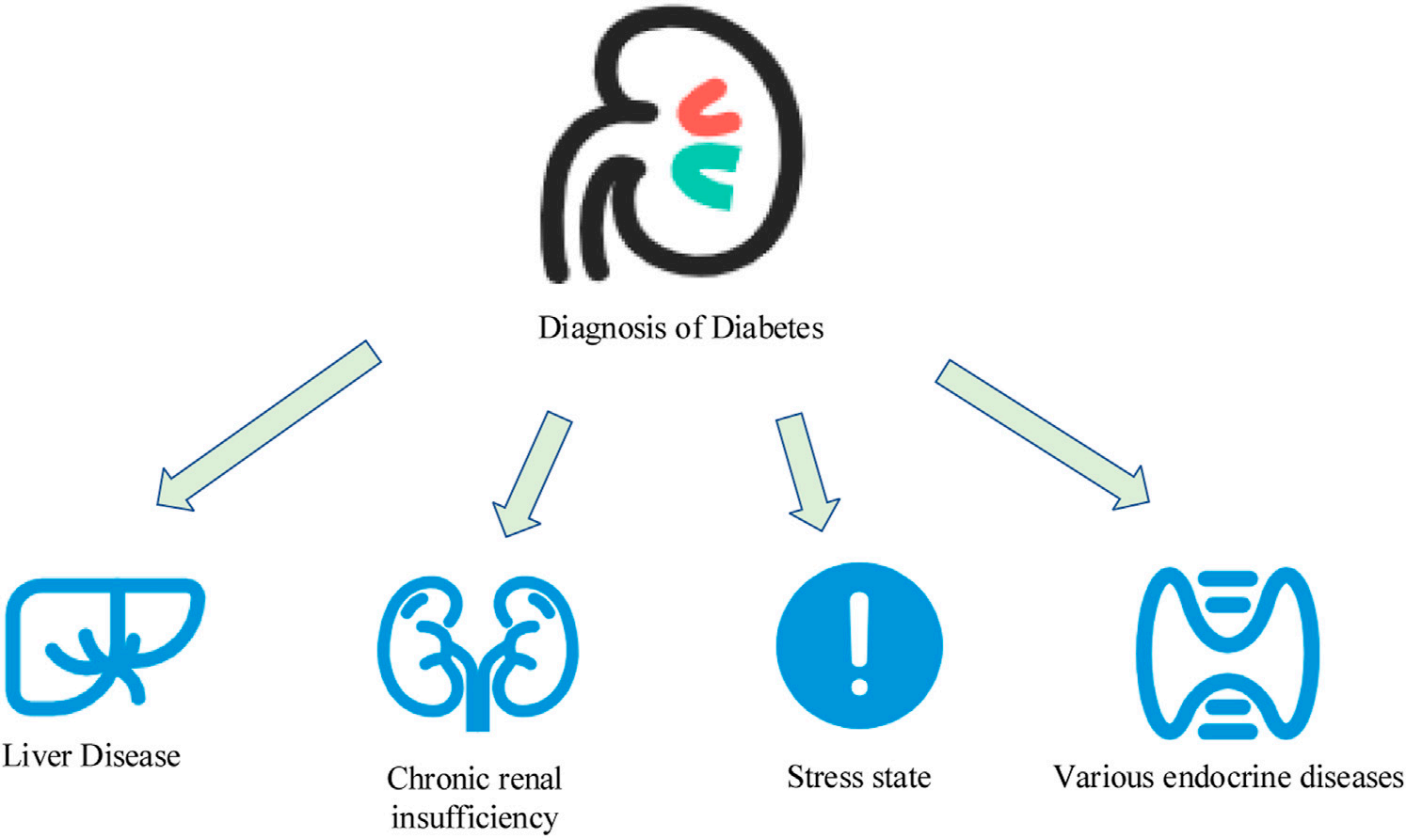
Diagnosis of diabetes.

Diabetes is a metabolic disease characterized by hyperglycemia. Hyperglycemia is caused by insufficient secretion of insulin and its biological function is impaired (Johal et al., 2017). Diagnosing diabetes is generally not difficult. The diagnosis can be made if the fasting blood glucose is greater than or equal to 7.0 mmol/L, or greater than or equal to 11.1 mmol/L within 2 h after a meal. The diagnosis is divided into type 1 and type 2 diabetes. The differential diagnosis of diabetes is shown in Figure 2.

#### 2.2.1 Liver disease

Patients with liver cirrhosis usually have abnormal glucose metabolism, which is usually fasting or hypoglycemia, and the blood sugar would rise rapidly after meals. Patients with prolonged illness, fasting blood sugar would also increase.

#### 2.2.2 Chronic renal insufficiency

There would be a slight abnormal glucose metabolism. Patients need to use some medications under the guidance of medical professionals to protect the kidney and repair the glucose metabolism function.

#### 2.2.3 Stressed state

Under stressful conditions, such as heart, cerebrovascular accident, acute infection, trauma, etc., blood sugar would be excessively increased, and it would return to normal within 1–2 weeks after the stress factor disappears (Sorli et al., 2017).

#### 2.2.4 Various endocrine diseases

Glucagonoma is a secondary cause of diabetes mellitus and has other characteristics besides hyperglycemia.

### 2.3 Concepts related to pancreas

In the upper abdomen of the human body, there is a small organ that is difficult to find, and that is the pancreas. Although the pancreas is small, it is very powerful and is one of the most important parts of the human body. The pancreas is a gland with endocrine and endocrine functions, and its physiological function and pathological changes are closely related to human health.

The pancreas is located retroperitoneally. In terms of secretion, although the pancreas is small, it contains many endocrine cells. These cells are also adjusting the physiological functions of the body during the process of digestion and absorption. If these cells change and secrete too much or too little, disease can result (Mario et al., 2017). The pancreas produces various digestive enzymes and insulin in the body to help the body break down proteins and other substances and lower blood sugar. Pancreatic juice is an exocrine substance that mainly includes alkaline bicarbonate and various digestive enzymes.

## 3 Pancreatic tissue as a target to treat diabetes signaling pathway

**FIGURE 3:**
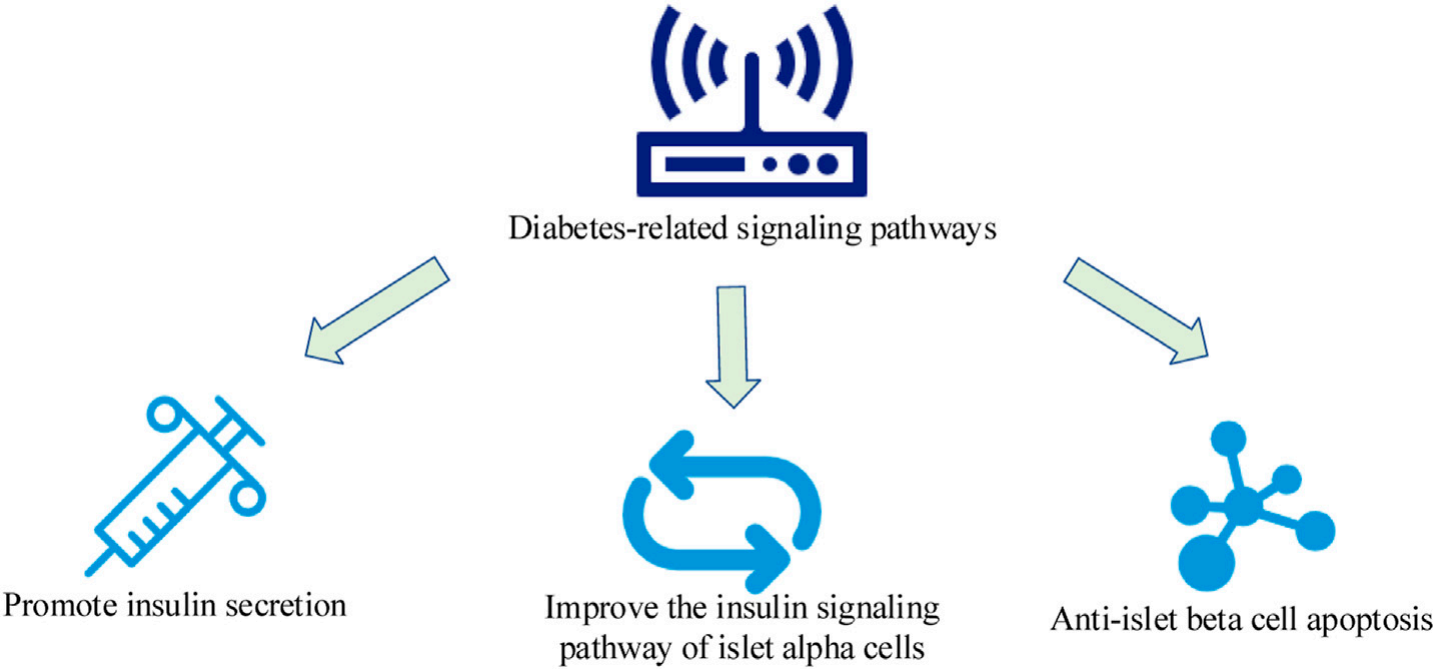
Pancreatic tissue as a target for the treatment of diabetes-related signaling pathways.

Diabetes is an endocrine inflammatory disease, and its clinical manifestations are multi-channel and multi-target regulation. The pancreas is the main lesion of diabetes, and it is also an important target for the treatment of diabetes. Its main functions are to promote the synthesis of hepatic glycogen and myoglycogen, the absorption and activation of glucose, and the inhibition of sugar xenobiogenesis. Figure 3 shows the relevant signaling pathways targeting pancreatic tissue.

### 3.1 Promotion of insulin secretion

#### 3.1.1 Calcium atom channels and ATP-sensitive potassium channels

The cells in the pancreas that produce and release the hormone insulin are endocrine cells with electrical excitation. Its secretion is mainly due to an increase in intracellular calcium concentration, which allows it to produce insulin. L-type calcium channels are the main channel for glucose-induced insulin. Its variation may cause type 2 diabetes, and the state of energy metabolism in the body also affects the electrical activity of sensitive potassium channels. Under physiological conditions, glucose enters the pancreas to produce energy from cellular metabolism that produces and releases the hormone insulin. Adenosine triphosphate closes sensitive potassium channels in the cell membrane, opening calcium channels in the cell, thereby activating cells in the pancreas that produce and release the hormone insulin to secrete insulin (Kiran et al., 2017). Therefore, calcium channels and sensitive potassium channels may be effective drug targets for type 2 diabetes.

#### 3.1.2 β-cell GLP-1 receptor signaling pathway

Glucagon-like peptide-1 (GLP-1) is a glucose concentration-based polypeptide hormone. It can regulate the gene expression, synthesis and secretion of cells in the pancreas that produce and release the hormone insulin, and can promote insulin secretion and inhibit insulin cell apoptosis. GLP-1 receptors are mainly distributed in the pancreas. After GLP-1 binds to its receptor, it can activate adenylate cyclase, thereby increasing intracellular cyclic adenylate and activating downstream protein kinases. GLP-1 activates the calcium atom signaling pathway through the acidity coefficient and cyclic adenylate binding protein pathway to promote the release of insulin.

### 3.2 Improvement of insulin signaling pathway in pancreatic islet cells by reducing glucagon secretion

Glucagon is a polypeptide hormone produced by pancreatic islet cells, and its abnormal secretion and metabolism may be related to the pathogenesis of type 2 diabetes. Under physiological conditions, cells in the pancreas that produce and release the hormone insulin block alpha cell glucagon production by paracrine (Arnold et al., 2018). The insulin receptor substrate, phosphatidylinositol kinase, is also expressed on cells, and insulin inhibits the gene expression of glucagon in islet cells through signal channels. In conclusion, the improvement of insulin resistance and the reduction of glucagon production is a new therapeutic approach.

### 3.3 Anti-islet beta cell apoptosis

The MAPK (mitogen-activated protein kinase signaling) pathway has a role in regulating cell growth, differentiation and apoptosis in mammalian cells. Different MAPK signaling pathways can be utilized by different extracellular stimuli, and their regulation can modulate various cellular responses. Stress-activated protein kinases and cell signaling cascades have important roles in cellular stress and cellular inflammatory factors. The results show that streptosporin can inhibit the occurrence of type 1 diabetes and reduce the phosphorylation level of its kinase, thereby reducing its upstream kinase activity.

## 4 Algorithm of diabetes under the precision medicine big data evaluation system

### 4.1 Diabetes risk input expression

The diabetes risk input expression is a model that combines the user’s risk index data (including past and present data). Since a patient has multiple medical records, multiple medical records appear in the same user’s medical records. Therefore, it is necessary to comprehensively consider the weight of each medical record, that is, the importance of the sub-medical record.

Definition 1: collection of risk indicators

It is assumed that user $G_{i}$ has lb sub-records (with time stamps), then all sub-records of $Q_{i}$ can form a set of risk indicators, and the formula is:

$$Q_{i}={\left\{\langle k_{ab},f_{ab}\rangle \right\}}_{b\in \left\{1,2,\ldots ,lb\right\}}$$ (1)

Among them, $k_{ab}$ is the time, and $f_{ab}$ is the feature vector of each index component; the entire data set can be represented as $Q={\left\{Q_{i}\right\}}_{a\in \left\{1,2,\ldots ,n\right\}}$ , and n is the number of patients.

For the b -th record $k_{ab},f_{ab}$ in $Q_{i}$ , its decay weight formula is:

$$Ph\left(b\right)=e^{-\infty b}/\overset{l-1}{\underset{k=0}{\sum }}e^{-\infty k}$$ (2)

Among them, ∞ is the adjustment weight range, and l is the time span.

Definition 2: risk input expression

The weighted average of each inspection record of 1 $G_{i}$ based on the decay weight can be extracted as an input expression, which is the risk input expression:

$$x_{a}=\overset{l_{a}}{\underset{b=1}{\sum }}Ph\left(l_{a}-b\right)f_{ab}$$ (3)

It can be obtained that the risk index set ${\left\{\langle k_{ab},f_{ab}\rangle \right\}}_{b\in \left\{1,2,\ldots ,la\right\}}$ of user $G_{i}$ can be normalized in the form of a matrix into the form of vector $x_{a}$ , which can be used as the standard input of the calculation model.

The model is based on the support vector machine algorithm. Parameter r,g represent function $f\left(x\right)$ , and its calculation method:

$$f\left(x\right)=rt+g$$ (4)

In the hyperplane, all the diabetes sample spaces are divided into two groups: one with diabetes and one without diabetes. Through mathematical transformation, it is the solution target:

$$\min_{r,g,\zeta }=\frac{1}{2}r^{T}r+C\overset{m}{\underset{k=1}{\sum }}\zeta^{k}$$ (5)

$$y_{a}\left(r^{T}\phi \left(x_{a}\right)+g\right)\geq 1,a=1,\ldots ,m$$ (6)

m is the number of training samples, and $y_{a}$ is the class label.

Definition 3: diabetes index

The Diabetes Risk Index (DI) represents the risk of developing diabetes and has a value in the range [0,1]. The corresponding DI value can be obtained by probabilistic correction of the value of the judgment function. Probabilistic calibration is performed:

$$R\left(y=1\left|x\right.\right)=1/\left(1+\exp\left(Sd\left(x\right)+F\right)\right)$$ (7)

Among them, S,F are conversion parameters.

DI is calculated as:

$$DI\left(x\right)=R\left(y=1\left|x\right.\right)$$ (8)

### 4.2 Diabetes risk model based on genetic factors

The inheritance of diabetes is determined by the relatedness of family members, so a suitable indicator should be used to measure the genetic association of two people.

#### 4.2.1 Genetic coefficient

In kinship, the blood of the child is inherited from the parents, so the genetic factor between the parent and the child is 1/2. Since the genetic factors represented by each side are all 1/2, the calculation formula of the genetic factors of the direct genetic relationship can be obtained:

$$k\left(X,Y\right)={\left(1/2\right)}^{M}$$ (9)

Through the calculation of the direct genetic relationship, the paragenetic genetic factors can be obtained. First, it is necessary to find out the most recent common ancestors $N_{1}$ and $N_{2}$ of members X and Y , and calculate the genetic coefficients of X,Y and each ancestor separately. After that, the sum is done to get the heritability coefficients of X and Y :

$$k\left(X,Y\right)=\overset{2}{\underset{i=1}{\sum }}k\left(X,N\right)k\left(Y,N\right)$$ (10)

In Formula (10), n is the total number of diabetes-positive family members of user R .

#### 4.2.2 Dynamic blood glucose expression

Ambulatory blood glucose sequence $g=\left(g_{1},g_{2},\ldots g_{n}\right)$ represents a time series of blood glucose values. The data $y\left(h\right)$ at time h can be predicted from the blood glucose value at time h−1,h−2,…h−n , and there is a complex non-linear relationship $f\left(\cdot \right)$ between them, which is expressed by Formula (12):

$$y\left(h\right)=f\left(y\left(h-1\right),y\left(h-2\right)\right),\ldots ,y\left(h-n\right)$$ (12)

It is assumed that the number of visible units and hidden units are m and n , respectively, then $z=\left(z_{1},z_{2},\ldots z_{m}\right)$ , and $x=\left(x_{1},x_{2},\ldots x_{n}\right)$ . b represents the bias of the visible layer, and c represents the bias of the hidden layer. The energy equation of the model is:

$$W\left(z,x\left|\theta \right.\right)=-bz^{T}-cx^{T}-vwx^{T}$$ (13)

It is expressed in components as:

$$W\left(z,x\left|\theta \right.\right)=-\overset{m}{\underset{i=1}{\sum }}b_{i}z_{i}-\overset{n}{\underset{i=1}{\sum }}c_{j}x_{j}-\overset{n}{\underset{j=1}{\sum }}\overset{m}{\underset{i=1}{\sum }}z_{i}W_{ij}x_{j}$$ (14)

The formula for calculating the joint probability distribution of the visible layer and the hidden layer is:

$$T\left(z,x\left|\theta \right.\right)=\frac{1}{B\left(\theta \right)}e^{-w\left(z,x\left|\theta \right.\right)}$$ (15)

$$B\left(\theta \right)=\underset{z,x}{\sum }e^{-w\left(z,x\left|\theta \right.\right)}$$ (16)

The marginal probability distribution of ambulatory blood glucose sequence z to x is:

$$T\left(z\left|\theta \right.\right)=\frac{1}{B\left(\theta \right)}\sum xe^{-w\left(z,x\left|\theta \right.\right)}$$ (17)

When the visible layer unit state is given, the activation probability of the $x_{i}$ -th hidden layer unit $z_{i}$ is solved as:

$$T\left(x_{i},\left|z_{i},\theta \right.\right)=sigm\left(b_{j}+\overset{m}{\underset{i=1}{\sum }}x_{i}W_{ij}\right)$$ (18)

Since cells are bidirectional, visible cells can be activated through hidden cells. The calculation method of the activation probability of the i -th visible unit $z_{i}$ is:

$$T\left(z_{i},\left|x,\theta \right.\right)=sigm\left(c_{j}+\overset{m}{\underset{i=1}{\sum }}W_{ij}x_{i}\right)$$ (19)

By experimenting with predictive models and evaluating them predictively, the formula for calculating the root mean square error is:

$$RMSE=\sqrt{\frac{\sum_{a=1}^{n}{\left(x_{a}-{\hat{x}}_{a}\right)}^{2}}{n}}$$ (20)

In Formula (20), $x_{a}$ is the predicted value of the a -th model; ${\hat{x}}_{a}$ is the actual value of the a -th model, and n is the capacity of the sample set.

## 5 Experimental exploration of the pancreas as the target for the treatment of diabetes under the precision medicine big data evaluation system

### 5.1 Experimental method

Under the precision medicine big data analysis system, this paper studied the signaling pathway of treating diabetes by targeting the pancreas. City A was selected as the research object, and the research was carried out from five aspects: the age structure of diabetes mellitus in 2015–2018, the blood sugar control standard of type 2 diabetes in the elderly, the change in the number of diabetic patients, the proportion of patients using pancreatic types, and the proportion of patients using pancreatic types.

### 5.2 Experimental analysis

#### 5.2.1 Age structure of diabetes

**FIGURE 4:**
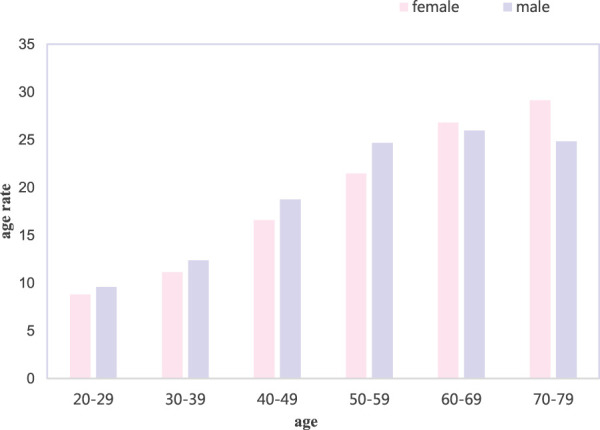
Age structure of diabetes.

The probability of disease in each age stage is different, and the age structure of diabetic patients in city A was investigated. The results are shown in Figure 4.

In the age structure of diabetes shown in Figure 4, the higher the female age, the greater the probability of developing diabetes. Men were more likely to develop diabetes at the age of 60–69, and there were more patients with diabetes over the age of 50. It can be seen that age is closely related to the occurrence of diabetes.

#### 5.2.2 The blood sugar control standard of type 2 elderly diabetes mellitus

Older people often experience symptoms of hypoglycemia. In addition, elderly diabetic patients are prone to arteriosclerosis and cardiovascular disease. Hypoglycemia can lead to complications such as cerebral infarction and myocardial infarction. The blood sugar control standards for type 2 elderly diabetes mellitus are shown in Table 1.

**TABLE 1:** Glycemic control criteria for type 2 diabetes in the elderly.

Health status	Reasonable saccharification (%)	Fasting or preprandial blood glucose (oI/L)	Blood sugar before bed (I/L)
healthy	<7.5	5.0–7.2	5.0–8.3
moderate health	<8.0	5.0–8.3	5.6–10.0
poor health	<8.5	5.6–10.0	6.1–11.1

For elderly diabetic patients, the efficacy and risk should be comprehensively considered, and the main purpose is to improve their quality of life. In addition, personalized blood glucose control indicators should be developed to improve the health of the elderly.

#### 5.2.3 Changes in the number of diabetic patients

**FIGURE 5:**
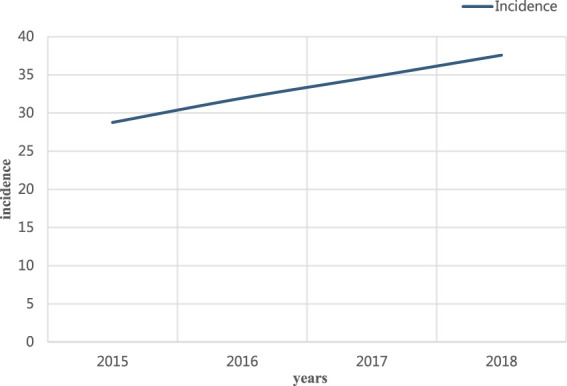
Number of people with diabetes.

Changes in the number of diabetic patients as the research subject, the number of diabetic patients in city A was investigated from 2015 to 2018. The specific results are shown in Figure 5.

Among the changes in the number of diabetic patients shown in Figure 5, the ratio of the number of diabetic patients showed an upward trend from 2015 to 2018. Among them, the proportion of diabetic patients in 2015 was about 28.76%; in 2018, the proportion of diabetic patients was about 37.57%, with an increase of 8.81%. It can be seen that in life, people should maintain a good diet and routine, and exercise regularly. With regular checkups, people can reduce the chances of developing diabetes.

#### 5.2.4 Ratio of patients using pancreatic species

**FIGURE 6:**
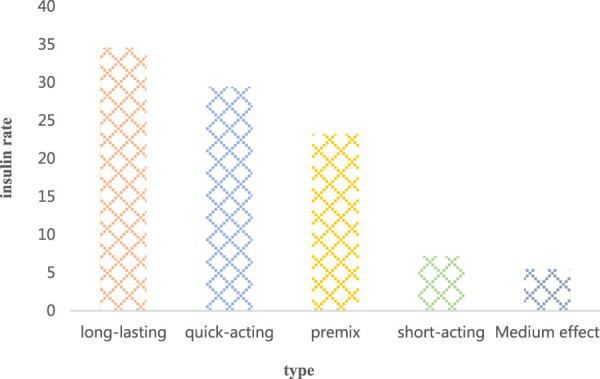
The proportion of patients using insulin type.

The main body of the investigation was the proportion of patients using pancreatic types in area A, and studies were carried out from long-acting, quick-acting, premixed, short-acting, and intermediate-acting. The results are shown in Figure 6.

In the proportion of patients using pancreatic types shown in Figure 6, more patients used pancreatic long-acting, fast-acting and premixed, and about 34.58% used long-acting patients; there was about 29.42% of the patients used quick-acting, and 23.25% of the patients used premix. The number of patients using pancreatic short-acting and intermediate-acting was relatively small; the number of patients using short-acting was about 7.21%, and the number of patients using intermediate-acting was about 5.54%.

#### 5.2.5 Changes in blood sugar using the pancreas

**FIGURE 7:**
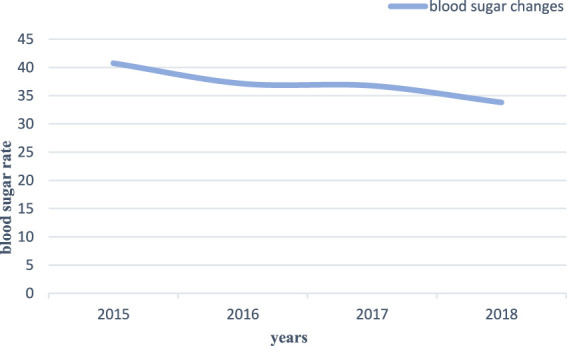
Blood glucose changes using the pancreas.

The pancreas affects glucose metabolism in the human body and plays an important role in diabetes. Figure 7 shows the results of the research content of the pancreas-targeted treatment of diabetic blood sugar changes.

In the pancreas-targeted treatment of diabetes shown in Figure 7, blood glucose rates continued to decline from 2015 to 2018. In 2015, the blood sugar rate of pancreas-targeted treatment of diabetes was about 40.75%; in 2018, the blood sugar rate of pancreatic-targeted treatment of diabetes was about 33.81%, with a decrease of 6.94%. It can be seen that the pancreas would secrete insulin, which can regulate the level of glucose in the blood and brings blood sugar back to normal.

## 6 Conclusion

Through the analysis and sharing of precision medicine big data, it would help the development of current medical technology and improve the level of medical treatment. This paper systematically discussed the diabetes signaling pathway targeting the pancreas, which has certain reference value for guiding the development of new drugs and the use of a variety of clinical drugs. The pancreas is mainly used to treat diabetes, and its main function is to stimulate pancreatic secretion and inhibit the release of glucagon. Apoptosis of the pancreas is an important factor affecting the amount of insulin secreted, and apoptosis of the pancreas is related to many signaling pathways. Therefore, the signal pathway related to diabetes explored from the perspective of the pancreas has a certain guiding effect on the development and clinical treatment of new drugs for diabetes.

## Data Availability

The original contributions presented in the study are included in the article/Supplementary Material, further inquiries can be directed to the corresponding author.
